# Cytotoxicity of psammaplin A from a two-sponge association may correlate with the inhibition of DNA replication

**DOI:** 10.1186/1471-2407-4-70

**Published:** 2004-09-30

**Authors:** Yahong Jiang, Eun-Young Ahn, Seung Hee Ryu, Dong-Kyoo Kim, Jang-Su Park, Hyun Joo Yoon, Song You, Burm-Jong Lee, Dong Seok Lee, Jee H Jung

**Affiliations:** 1 Department of Chemistry and Biohealth Product Research Center, Inje University, Kimhae 621-749, S Korea; 2 School of Pharmaceutical Engineering, Shenyang Pharmaceutical University, Shenyang 110016, China; 3 Department of Chemistry, Pusan National University, Pusan, 609-735, S Korea; 4 Department of Microbiology, Inje University, Kimhae 621-749, S Korea; 5 Department of Medical Laboratory Science, Inje University, Kimhae 621-749, S Korea; 6 College of Pharmacy, Pusan National University, Pusan 609-735, S Korea

## Abstract

**Background:**

SV40 DNA replication system is a very useful tool to understand the mechanism of replication, which is a tightly regulated process. Many environmental and cellular factors can induce cell cycle arrest or apoptosis by inhibiting DNA replication. In the course of our search for bioactive metabolites from the marine sponges, psammaplin A was found to have some anticancer properties, the possible mechanism of which was studied.

**Methods:**

Cell viability was determined by Cell Counting Kit-8 (CCK-8) to count living RAW264.7 cells by combining 2-(2-methoxy-4-nitrophenyl)-3-(4-nitrophenyl)-5-(2,4-disulfophenyl)-2H-tetrazolium (WST-8) and 1-methoxy-phenazine methosulfate (1-methoxy-PMS). The effect of psammaplin A on DNA replication was carried out in SV40 DNA replication system *in vitro*. The activities of topoisomerase I and polymerase α-primase were measured by the relaxation of superhelical plasmid DNA and the incorporation of [^3^H]dTTP to the template respectively. The ssDNA binding activity of RPA was assessed by Gel Mobility Shift Assay (GMSA).

**Results:**

We have found that psammaplin A delivers significant cytotoxic activity against the RAW264.7 cell line. It was also found that psammaplin A could substantially inhibit SV40 DNA replication *in vitro*, in which polymerase α-primase is one of its main targets.

**Conclusion:**

Taken together, we suggest that psammaplin A-induced cytotoxicity may correlate with its inhibition on DNA replication. Psammaplin A has the potential to be developed as an anticancer drug.

## Background

DNA replication in eukaryotic cells is a tightly regulated process [1]. The regulation of DNA replication is central to understanding the regulation of cell cycle and virus proliferation, events that have a direct impact on our understanding human disease. One critical component of cell cycle regulation is the initiation of DNA replication. The timing of initiation is precisely controlled and is sensitive to both environmental and cellular factors. If DNA replication is blocked by inhibitors or the template is damaged by radiation or other factors, signals are generated that can induce cell cycle arrest or apoptosis [2,3].

Much of what is currently known about the mechanism of DNA replication in eukaryotic cells has come from studying SV40 and related viruses. SV40 virus can use the host replication machinery for its own DNA replication together with the virally encoded SV40 T-antigen. SV40 T-Ag is a multifunctional regulatory protein with numerous biochemical activities, and it has been classified as a member of superfamily III helicase and can unwind dsDNA and RNA [4,5]. All other proteins are supplied by host cells. In replication, replication protein A (RPA) mediates unwinding of SV40 origin-containing DNA in the presence of SV40 T-Ag and the DNA polymerase α-primase complex (pol α-primase) [6,7], which is necessary for the initiation of SV40 DNA replication [8,9].

Psammaplin A is a symmetrical bromotyrosine-derived disulfide dimer that was originally isolated in 1987 from the *Psammaplysilla *sponge [10]. Early studies revealed that psammaplin A had general antibacterial and antitumor properties. In 1999, it was found that psammaplin A exhibited significant *in vitro *antibacterial activity against both *Staphylococcus aureus *(SA) and methicillin-resistant *Staphylococcus aureus *(MRSA), which was inferred to be the result of induced bacterial DNA synthesis arrest by psammaplin A through inhibiting DNA gyrase [11]. Given the increasingly rapid emergence of multi-drug resistant bacterial strains and the corresponding threat to public health, there is significant interest in the development of structurally novel antibacterial agents such as psammaplin A. Additionally, psammaplin A has been reported to exhibit certain inhibition of a number of enzymes including topoisomerase II (topo II) [12], farnesyl protein transferase [13], leucine aminopeptidase [13], and latest reported chitinase [14]. Among these enzymes, topo II, as one required protein for eukaryotic DNA replication, as well as bacterial DNA gyrase belongs to the topoisomerase family of enzymes responsible for the remolding of DNA topology. Since psammaplin A can inhibit bacterial DNA synthesis through DNA gyrase inhibition, and much of the basic enzymology of the eukaryotic replication fork has close homologies with its prokaryotic counterpart, we wonder whether psammaplin A also can induce eukaryotic DNA replication arrest or not.

We have reported that psammaplin A displayed significant cytotoxicity against human lung (A549), ovarian (SK-OV-3), skin (SK-MEL-2), CNS (XF498), and colon (HCT15) cancer cell lines [15]. In this paper, psammaplin A was found to have dose-dependent cytotoxicity on macrophage cell line. In order to clarify the possible mechanism of the cytotoxicity and also verify our conjecture of its possible action on DNA replication, the effect of psammaplin A on eukaryotic DNA replication was examined by using *in vitro *SV40 DNA replication system. According to our result that psammaplin A can induce eukaryotic DNA replication arrest through inhibiting some important replication proteins, we suggest that psammaplin A-induced cytotoxicity may correlate with its inhibition on DNA replication, and one of the main target molecules could be DNA polymerase α-primase.

## Methods

### Psammaplin A, proteins, cell extracts and DNA

Psammaplin A sample was a gift from a Dr. Jung's lab of Pusan National University. SV40 origin-containing circular duplex DNA (pUC-ori^+^), SV40 T-Ag, topoisomerase I (topo I), human DNA polymerase α-primase (pol α-primase), replication protein A (RPA), and HeLa extract were prepared as described previously [16].

### Cell lines and chemicals

Media for cell culture including HY, DMEM and RPMI were purchased from the Sigma Chemical Co. (St. Louis, MO, USA) and Fetal Calf Serum (FCS) was from Gibco-BRL (Gaithersburg, MD, USA). Cell Counting Kit-8(CCK-8) was purchased from Dojin Laboratories (Kumamoto, Japan). The mouse macrophage cell line RAW264.7 was purchased from Korean Cell Line Bank (Seoul, Korea).

### Cell viability assay

Cell viability was determined by CCK-8 to count living cells by combining WST-8 and 1-Methoxy PMS [17]. Briefly, macrophage cells (RAW264.7) were seeded into 96 well plates at an initial density of 10^5 ^cells/well. After incubation with the indicated concentrations of psammaplin A for 12 hr, 10 μl of kit reagent was added and incubated for a further 3 hr. Cell viability was obtained by scanning with a microplate reader at 450 nm.

### SV40 DNA replication in vitro

The reactions were carried out as described previously [18]. In brief, the reaction mixtures (40 μl) included 40 mM creatine phosphate-di-Tris salt (pH 7.7), 1 μg of creatine kinase, 7 mM MgCl_2_, 0.5 mM DTT, 4 mM ATP, 200 μM UTP, GTP, and CTP, 100 μM dATP, dGTP, and dCTP, 25 μM [^3^H]dTTP (300 cpm/pmol), 0.6 μg of SV40 T-Ag, 0.23 μg of pUC-ori^+^, HeLa extracts, and psammaplin A as indicated. The reactions ran at 37°C for 2 hr, after which the acid-insoluble radioactivity was measured [18].

### Topo I assay

Topo I was measured by the relaxation of superhelical plasmid DNA [19]. The 20 μl assay mixture contained 50 mM Tris-HCl (pH 7.5), 120 mM KCl, 10 mM MgCl_2_, 0.5 mM DTT, 0.5 mM EDTA, bovine serum albumin (30 μg/ml), pUC118 (20 μg/ml), topo I (1 unit), and various amount of the psammaplin A. After 30 min at 30°C, the reactions were stopped by the addition of 5 μl of 5% NaDodSO_4_/25% (wt/vol) Ficoll 400 (Pharmacia) containing 0.25 mg of bromophenol blue per ml. The samples were then loaded onto the agarose gel (0.8%) for electrophoresis followed by photography.

### ssDNA binding assay

The assay was performed according to the published procedures [7]. The reaction mixture (20 μl) contained 50 mM Hepes-KOH (pH 7.5), 150 mM NaCl, 1 mM MgCl_2_, 0.5 mM DTT, 10% glycerol, 50 fmol of 5'-^32^P-labeled oligo(dT)_50 _(2200 cpm/fmol), plus the indicated amount of RPA, and was incubated for 15 min at room temperature. The complex was electrophoretically separated on a 5% polyacrylamide gel in 0.5 × TBE (89 mM Tris borate, 2 mM EDTA) at 15 V/cm. The gel was then dried and exposed to X-ray film.

### Pol α-primase assay

DNA pol α-primase activities were assayed as described previously [20] with the following modifications. Reaction mixtures (30 μl) contained 40 mM creatine phosphate/di-Tris salt, pH 7.7, 1.0 μg of creatine kinase, 7 mM MgCl_2_, 0.5 mM DTT, 6 μg of bovine serum albumin, 4 mM ATP, 33 μM of [^3^H]dTTP (500 cpm/pmol), 0.1 μg of poly (dA)_4500_: oligo (dT)_25 _(20:1), DNA pol α-primase, and psammaplin A as indicated. After incubation at 37°C for 30 min, acid-insoluble radioactivity was determined [18].

### Statistical analysis

Values are presented as mean ± SD. Data was initially analyzed by one-way analysis of variance (ANOVA) and comparison of groups was made using Turkey test (SPSS software).

## Results

### Effect of psammaplin A on the viability of macrophage cell line

**Figure 1:**
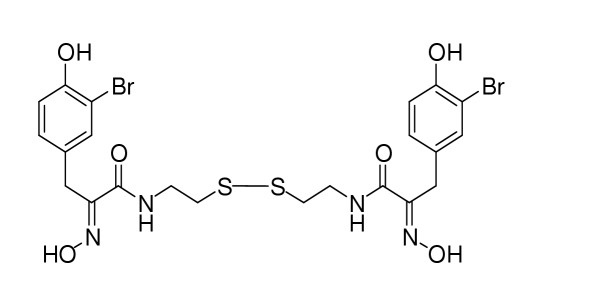
The structure of psammaplin A.

**Figure 2:**
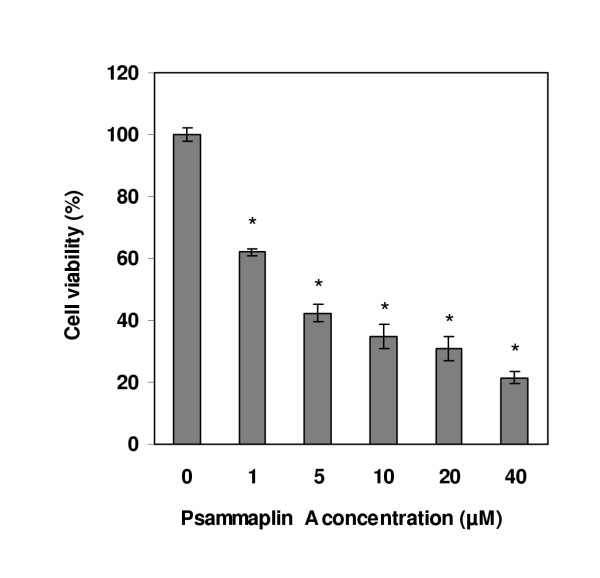
Effect of psammaplin A on the viability of macrophage cell line RAW 264.7. Macrophage cells were treated with various concentrations of psammaplin A for 12 hr. Relative cell viability was determined by WST-8 and 1-Methoxy PMS and is shown as a percentage of living cells. Data are shown as means ± SD of three independent experiments. * Data are significantly different from control group at *p *< 0.001.

As shown in Fig 1, psammaplin A is a symmetrical bromotyrosine-derived disulfide dimer, which exhibits *in vitro *antibacterial activity against methicillin-resistant *Staphylococcus aureus *(MRSA). Psammaplin A is rather interesting owing to its two identical domains which are linked through a disulfide bridge. Macrophage cells are one of the key players in the early innate immune response, and they release inflammatory chemicals known as cytokines when they are activated. This sort of inflammation is not always a good thing, and overactive macrophage cells have been implicated in a number of human diseases, including arthritis and sepsis. When we studied the effect of psammaplin A on the viability of macrophage cell line RAW264.7, a reduced cell count was observed in the psammaplin A-treated cells and this decrease in the number of living cells also showed good dose-dependent (Fig 2).

### Inhibition of SV40 DNA replication in vitro by psammaplin A

**Figure 3:**
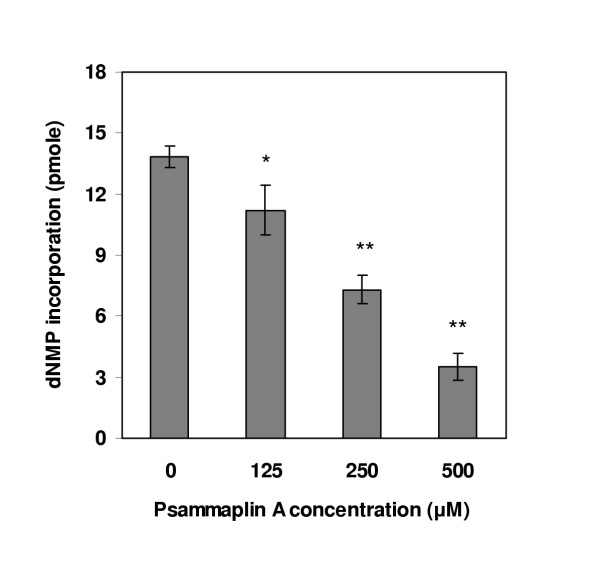
Effect of psammaplin A on SV40 DNA replication *in vitro*. Replication reaction comprised SV40 origin-containing DNA (pUC-ori^+^), SV40 T-Ag, HeLa cytosolic extract (100 μg), [^3^H]dTTP, and the indicated amounts of psammaplin A. Reaction mixtures were incubated at 37°C for 2 hr, after which the acid-insoluble radioactivity were measured. Data are shown as means ± SD of three independent experiments. Data are significantly different from control group at *p *< 0.05 (*)and at *p *< 0.001 (**).

As it has been mentioned in the background, we wonder that psammaplin A has inhibitory effect on eukaryotic DNA replication or not. To verify this conjecture and also clarify the possible mechanism of psammaplin A-induced cytotoxicity, we examined the effect of psammaplin A on DNA replication using an *in vitro *SV40 DNA replication system. Addition of increasing amounts of psammaplin A quantitatively inhibited SV40 DNA replication with HeLa cytosolic extract (Fig 3).

**Figure 4:**
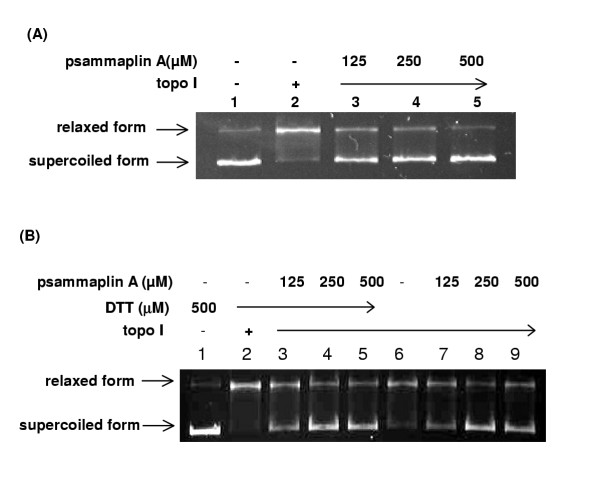
Psammaplin A inhibited topo I catalytic activity. Topo I activity was measured by the relaxation of superhelical plasmid DNA. The assay mixture (20 μl) contained pUC118 (20 μg/ml), topo I (1 unit), and various amounts of the psammaplin A. After 30 min at 30°C, the reactions were stopped by the addition of 5 μl of stop solution. The samples were then loaded onto the agarose gel (0.8%) for electrophoresis followed by photography. (A) The reaction mixtures contain 0.5 mM DTT; (B) Compare the reactions in the presence and absence of 0.5 mM DTT.

Inhibition of replication by psammaplin A in a cell-free system could be mediated either by damaging the template or by modulating the activity of a protein (or proteins) that is required for replication. The former mechanism is unlikely, because we have directly checked the effect of psammaplin A on DNA and didn't find any detectable damages to the template (data not shown). In order to find what proteins in DNA replication were affected by psammaplin A, we checked topo I activity at first, which plays key roles in DNA replication, transcription, and recombination by forming transient DNA single-strand breaks and acting as DNA strand transferase. In addition, the topoisomerase is now considered to be an important cancer chemotherapeutic target. The inhibitory effect of psammaplin A on the catalytic activity of topo I was shown in Fig 4a. The plasmid DNA was in the superhelical form (lane 1), and topo I relaxed the supercoiled DNA (lane 2). Psammaplin A inhibited the relaxation by topo I strongly at a concentration of 125 μM.

Nicolaou and his colleagues reported that the DTT present in many enzyme assays could reduce the disulfide bond of psammaplin A to the corresponding free thiol [24]. In their experiment without DTT, psammaplin A exhibited no detectable inhibition of bacterial DNA gyrase up to 100 μg/ml. They suggested the weak inhibitory activity observed by the earlier authors could be attributed to the presence of the free thiol rather than the product itself. In our experiments detecting the effect of psammaplin A on topo I, no difference was found in the same gel between the reactions in the presence and absence of 0.5 mM DTT (Fig 4b).

**Figure 5:**
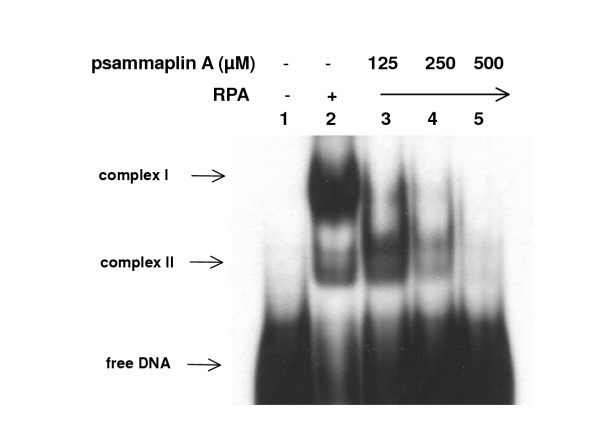
Psammaplin A inhibited ssDNA binding activity of RPA. Indicated amount of either human RPA or a mixture of both RPA and psammaplin A were combined with ^32^P-labeled oligo(dT)_50 _and incubated for 15 min at room temperature. The protein-DNA complexes were then separated from unbound DNA by 5% polyacrylamide (acrylamide:bisacrylamide, 29:1) gel electrophoresis.

In replication, RPA mediates unwinding of SV40 origin-containing DNA in the presence of SV40 T-Ag and topo I. It interacts with SV40 T-Ag and the DNA pol α-primase complex, which is necessary for the initiation of SV40 DNA replication [8,9]. Here, we examined the effect of psammaplin A on RPA's ssDNA-binding activity. As shown in Fig 5, RPA formed stable complexes with oligo(dT)_50_, which appeared as two distinct bands in the polyacrylamide gel. The ssDNA binding activity of RPA was inhibited by psammaplin A in a concentration-dependent manner, and 500 μM of psammaplin A totally inhibited the ssDNA-binding activity of RPA.

**Figure 6:**
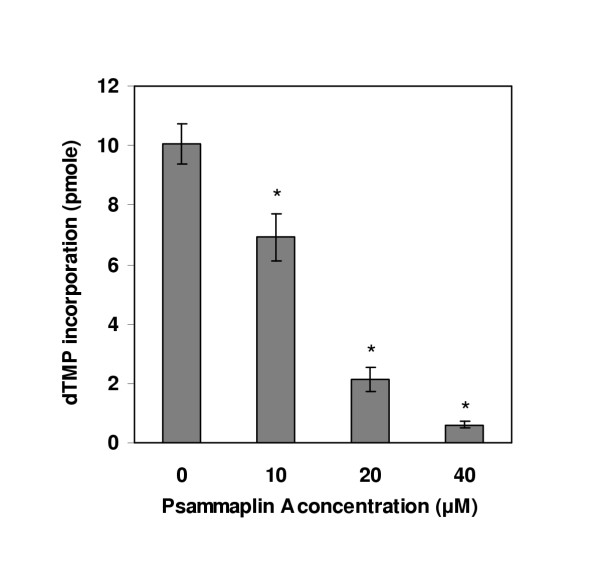
Effect of psammaplin A on pol α-primase activity. Indicated amounts of psammaplin A were added to the reaction mixtures, which included 0.1 unit of human pol α-primase complex, 4 mM ATP, 33 μM of [^3^H]dTTP, 0.1 μg of poly (dA)_4500_:oligo(dT)_25_(20:1), incubations were performed at 37°C for 30 min, followed by measurement of acid-insoluble radioactivity. Data are shown as means ± SD of three independent experiments. * Data are significantly different from control group at *p *< 0.001.

As described above, DNA pol α-primase complex is necessary for the initiation of SV40 DNA replication. To further investigate the inhibitory effect of psammaplin A in replication, we tested psammaplin A for inhibition of pol α-primase activity to see whether it's inhibitory effect on DNA replication correlate with pol α-primase activity. As shown in Fig 6, the activity of pol α-primase was inhibited by psammaplin A, and 40 μM of psammaplin A inhibited about 94% the activity of pol α-primase.

## Discussion

Psammaplin A has exhibited inhibition on general bacterium, some actinomycetes and fungi, and it also has cytotoxicity toward several cancer cell lines. In our research, we found that psammaplin A deliver significant cytotoxic activity against macrophage cell line RAW264.7. Which process is mostly affected by psammaplin A in cell cycle? What is the mechanism of the inhibition? Enlightened by the inhibitory effects of psammaplin A on bacterial DNA synthesis, bacterial DNA gyrase and eukaryotic topo I, we investigated the effect of psammaplin A on DNA replication using SV40 replication *in vitro *system attempting to find out the target process and molecules of psammaplin A and have a glimpse on cell cycle regulation.

In our study, we found that psammaplin A inhibited SV40 DNA replication *in vitro*. In order to clarify the inhibition mechanism, further work were carried out. In SV40 DNA replication, three factors, SV40 T-Ag, RPA, and pol α-primase complex, are essential for initiation process. In the presence of topo I, SV40 T-Ag will continue to unwind the DNA to form a highly unwound DNA [21]. DNA synthesis with three factors and topoisomerase can be quite extensive [22]. We have suggested that psammaplin A might interfere with some molecules that are required to establish replication forks during the initiation reaction. To address this possibility, we asked whether psammaplin A inhibits topo I, RPA's ssDNA binding activity, and pol α-primase activity. In addition, the topo I is now considered to be important cancer chemotherapeutic target. In mammalian cells, actions of antitopoisomerase drugs on replication, transcription, and other processes ultimately activate pathways of programmed cell death [23]. Psammaplin A inhibited the DNA relaxation activity of topo I and the ssDNA binding activity of RPA in a dose-dependent manner, and up to 500 μM, psammaplin A can inhibit both the activities of topo I and RPA completely. On the other hand, psammaplin A significantly reduced pol α-primase activity at 40 μM. The above results indicate that major inhibition of SV40 DNA replication by psammaplin A may be due to the inhibition of pol α-primase activity. Here, we cannot rule out the possibility that psammaplin A inhibit the activity of SV40 T-Ag, because it is essential for initiation process.

It is puzzling that the DNA pol α-primase, RPA and topo I were readily inhibited by low concentration of psammaplin A whereas the SV40 DNA replication assay still showed about more than 80% DNA replication in the presence of 125 μM psammaplin A. In our opinion, at least three points could account for this discrepancy. First, the cell extract we have used to support *in vitro *DNA replication system includes a large number of proteins, while in topo I assay, RPA binding assay and pol α-primase assay, purified proteins were used. Many of the proteins in the crude extracts can affect each other by physical or functional interactions. For example, in the process of DNA replication initiation, RPA interacts with T-Ag and DNA pol α-primase, and it is believed that RPA can both stabilize the unwound DNA and stimulate DNA pol α. The universal protein-protein interactions in crude extracts make its working environment quite different from that of purified protein assay system. Second, even for the same protein, for example, pol α-primase, the concentration in the cell extract and in the purified pol α-primase assay is not comparable before any quantification of the replication proteins in the cell extract. Third, due to the active disulfide moiety in the structure of psammaplin A, it is possible that psammaplin A could interact with some particular cellular targets in the crude extract, which may lead to covalent modification of the biological targets and psammaplin A itself. Therefore, the free available concentration of psammaplin A in the crude extract might be different from that in purified protein assay system.

Different from the effect of psammaplin A on SV40 DNA *in vitro *replication, the significant inhibition of psammaplin A on the viability of macrophage RAW264.7 cells occurred at a relatively low concentration. There is the possibility that DNA replication might not be the single or primary event that affected by psammaplin A. It should be evident that the event of DNA replication in a living cell is more complicated than that in the crude extract system because of many existing cell cycle signals. So, it is still vague whether the ability of psammaplin A to inhibit cellular viability is correlated with its ability to inhibit DNA replication. In order to make it clear, it is necessary to check the effects of psammaplin A on other macromolecular synthesis (RNA synthesis and protein synthesis). Although the results in this paper do not clearly define the mechanism of cytotoxicity of psammaplin A, they have convincingly shown that psammaplin A possesses the abilities to inhibit DNA replication and some important replication proteins.

Because of the disulfide bridge linking two identical subunits in the structure of psammaplin A, in this study, we also paid attention to the potential reduction effect of DTT present in the topo I assay. Different from the report of Nicolaou [24], we didn't catch any difference between the reactions in the presence and absence of DTT. There exist two possibilities. One is that in our reaction system, DTT couldn't reduce psammaplin A to the corresponding free thiol. Comparing the *in vitro *assay conditions in this study, we can find that all these assays were performed in similar conditions (for example: pH 7.5~7.7, 0.5 mM DTT and Mg^2+ ^ion environment). Given the mildness and tolerance of these reaction mixtures, we guess that psammaplin A is stable in the assays. Of course there is the second possibility that psammaplin A was reduced in the reaction, but the reduction product had nearly the same inhibition effect on topo I as the original compound. Further investigation aimed at this question need to be carried out.

## Conclusions

Based on our results, we suggest that the cytotoxicity of psammaplin A might be related to the inhibitory effect it has on the fundamental cellular process-DNA replication, and one of the main target molecules of psammaplin A could be pol α-primase.

## Competing interests

The authors declare that they have no competing interests.

## Authors' contributions

YHJ and DKK conceived and designed the experiments and wrote the manuscript. YHJ performed all of the experiments. JHJ provided psammaplin A sample. EYA, SHR, JSP, HJY, SY, BJL and DSL participated in the conception, supervision, coordination and guidance of the study and manuscript preparation. All authors read and approved the final manuscript.

## Pre-publication history

The pre-publication history for this paper can be accessed here:
